# MicroRNA profiling in the left atrium in patients with non-valvular paroxysmal atrial fibrillation

**DOI:** 10.1186/s12872-015-0085-2

**Published:** 2015-08-29

**Authors:** Jiangang Wang, Shiqiu Song, Changqing Xie, Jie Han, Yan Li, Jiahai Shi, Meng Xin, Jun Wang, Tiange Luo, Xu Meng, Bo Yang

**Affiliations:** Department of Cardiac Surgery, Beijing Anzhen Hospital, Capital Medical University, Beijing, 100029 P.R. China; Department of Internal Medicine, Vidant Medical Center, Broody School of Medicine, East Carolina University, Greenville, North Carolina 27834 USA; Department of Cardiothoracic Surgery, Affiliated Hospital of Nantong University, Nantong, 226001 China; University of Michigan Cardiovascular Center, University of Michigan, Ann Arbor, Michigan 48109 USA

## Abstract

**Background:**

We aimed to identify the miRNA expression profiles in left atrial appendage, with the intention of identifying miRNAs that were significantly associated with non-valvular paroxysmal AF.

**Methods:**

The RNA samples were isolated from healthy controls (n = 5) and patients with atrial fibrillation (n = 8). To confirm the findings obtained by analyzing the miRNA profile, we measured the expression of selected miRNAs in the entire cohort by quantitative PCR.

**Results:**

Ten specific miRNAs were found to be differentially expressed between atrial fibrillation and healthy controls with more than a 2-fold change (P < 0.05). Consistent with the data obtained for the profile, expression levels of miRNA-155, miRNA-146b-5p and miRNA-19b were significantly increased in patients with atrial fibrillation. Interestingly, levels of miRNA-146b-5p and miRNA-155, which are known to be associated with inflammation, were independently and positively associated with left atrium dimension, atrial fibrillation duration and high sensitivity C-reactive protein levels. By using four Databases (TargetScan, miRanda, Starbase Clip-seq and miRDB) to perform target gene prediction, there were four genes were related to the inflammatory response and fibrosis, and three others encoding cardiac ion channel proteins. As a result of TaqMan qPCR and Western analysis, the relative mRNA and protein expression level of three target genes (DIER-1, TIMP-4 and CACNA1C) were significantly lower in the atrial fibrillation group than that in the healthy control group.

**Conclusions:**

Expression of inflammation-associated miRNAs is significantly up-regulated in the left atrial appendage of patients with non-valvular paroxysmal atrial fibrillation, which may play a significant role in electrical and structural remodeling.

**Electronic supplementary material:**

The online version of this article (doi:10.1186/s12872-015-0085-2) contains supplementary material, which is available to authorized users.

## Background

MiRNAs are endogenous ~23 nt RNAs that play important gene-regulating roles in animals and plants by pairing to the messenger RNAs (mRNAs) of protein-coding genes to direct their post-transcriptional regulation [1, 2]. Therefore, measuring miRNA expression can be useful for gene regulation studies at systems-level, especially when miRNA measurements are combined with mRNA profiling and other genome-scale data. Also, it is reported that miRNAs are unusually well-preserved in a range of biological specimens. This has led to considerable interest in the development of miRNAs as biomarkers for diverse molecular diagnostic applications, including in the treatment of cancer, cardiovascular diseases, autoimmune diseases and forensics [3–5]. Accordingly, miRNA profiling has become an area of interest for researchers and investigators working in various areas of biology and medicine [6].

Atrial fibrillation (AF) is the most common sustained cardiac rhythm disorder, and is increasing in prevalence and incidence [7, 8]. With the recent and rapid strides in the field of miRNA research, investigators have begun to appreciate the roles of mRNAs in the cardiovascular system [9]. Several studies have reported the direct involvement of miRNAs in controlling the cardiac excitability and arrhythmogenesis in specific pathophysiological conditions [10–12]. A group of miRNAs (such as miR-1 and miR-328) have been shown to regulate the genes encoding cardiac ion channel proteins and other relevant genes. Some of these miRNAs have been shown to be involved in AF, and some are considered to have the potential to regulate AF based on their target genes [13–15]. However, since most of the specimens have been obtained from patients with valvular AF, from previous studies one cannot distinguish whether the findings reveal inherent characteristics of the atria themselves or are due to underlying valvular heart diseases.

AF is as a complex electrical phenotype involving complex factors beyond electrophysiology. AF may have distinct underlying mechanisms, and different miRNAs might be involved in different types of AF. Currently, there is a growing body of evidence suggesting that a specifically altered pattern of miRNA expression in atrial tissue and plasma is associated with AF [16–20]. However, the miRNA signature in AF, particularly in non-valvular AF, is still unknown. Here we aimed to identify the miRNA expression profiles in left atrial appendage (LAA), with the intention of identifying miRNAs that were significantly associated with non-valvular paroxysmal AF (PAF).

## Methods

A detailed description of Methods is available in the Additional file 1.

## Results

### Study population

A total of 47 subjects were studied. LAAs were obtained from 30 patients with PAF. The mean age was 48.5 ± 7.2 years, and 63 % were males. The time since the first diagnosis of AF was ≈ 2 years. The mean left atrial diameter (LAD) was 53.1 ± 2.2 mm. The HC group consisted of seventeen dumped LAA samples obtained from transplant donors following heart transplantation. The HC group consisted of 13 males and 4 females; their mean age was 33 ± 1.3 years. The clinical characteristics of the 2 study populations are summarized in Additional file 2.

### MiRNA profiles in AF patients versus healthy controls

**Fig. 1:**
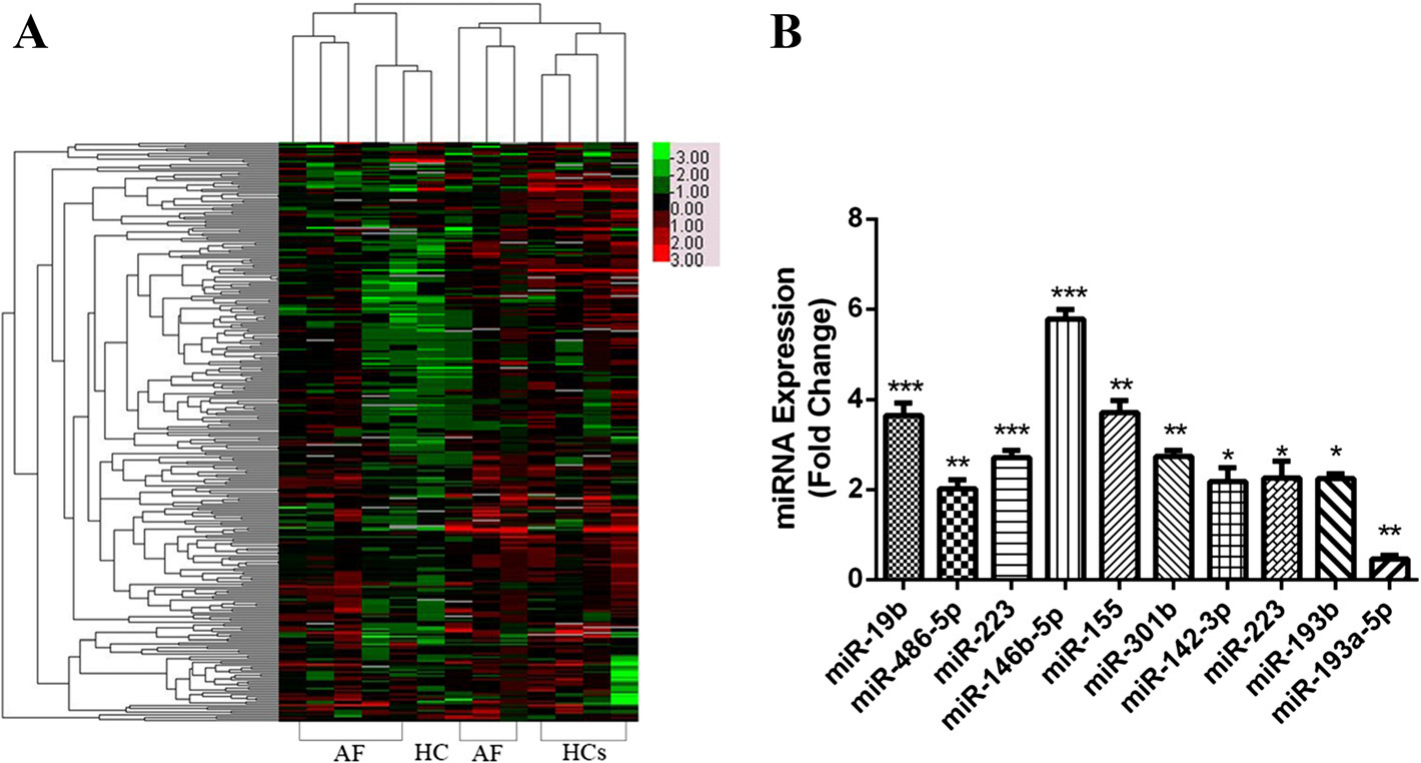
Profile of miRNAs in patients with atrial fibrillation versus health controls. **a** Heat map diagram t. **b** qRT-PCR verification of the miRNA expression profile in atrial fibrillation. ***P < 0.001; **P < 0.01; *P < 0.05

To determine the levels of miRNAs in LAA in patients with non-valvular PAF, we performed miRNA profiling in 8 patients with AF and in 5 HCs (Additional file 3 and Additional file 4). The miRNA expression profiles of AF and HCs were compared. The levels of miRNAs profoundly differed between patients and HCs, as illustrated in the heat map diagram shown in Fig. 1a. Quantification revealed that 10 miRNAs were differentially expressed between the patients and HCs with more than 2-fold change (*P* < 0.05). These miRNAs were miR-155, miR-146b-5p, miR-19b, miR-142-3p, miR-486-5p, miR-223, miR-193b, miR-519b-3p, miR-301b and miR-193a-5p.

The RT-PCR analysis confirmed the significant up-regulation of miR-155, miR-146b-5p, miR-19b, miR-142-3p, miR-486-5p, miR-223, miR-193b, miR-519b-3p and miR-301b, and significant down-regulation of miR-193a-5p (Fig. 1b). In particular, miR-155, miR-146b-5p, and miR-19b demonstrated the most pronounced changes among the 10 differentially expressed miRNAs. These qRT-PCR results confirmed the miRNA microarray results and indicated the potential roles of miRNAs in the progression of AF.

**Fig. 2:**
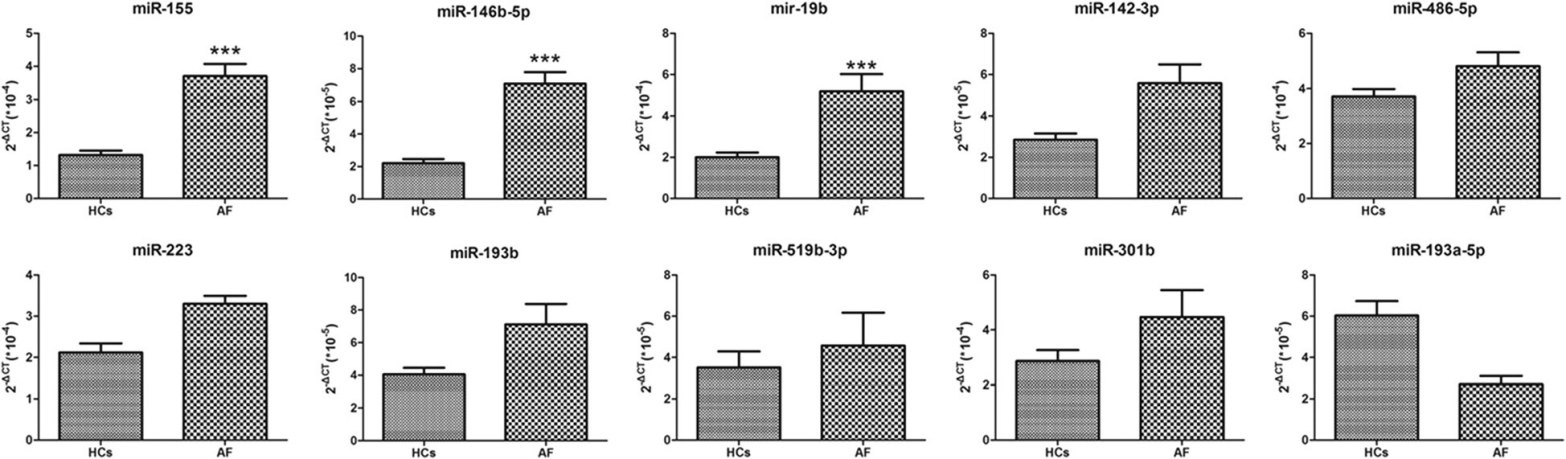
Validation of the differentially expressed miRNAs by qRT-PCR. Expression of selected miRNAs in left atrial antrum obtained from patients with atrial fibrillation (n = 30) and health controls (n = 17), as determined by TaqMan PCR. *** *P* < 0.001; ** *P* < 0.01; * *P* < 0.05

To confirm the findings of the miRNA profile analysis, we measured the expression of selected miRNAs in the entire cohort (n = 47) by using TaqMan quantitative PCR (qPCR). As shown in Fig. 2, the expression of predominantly inflammation-associated miRNAs such as miR-146b-5p, miR-19b, and miR-155 [21–23] were significantly increased in patients with AF.

### Correlation between miRNAs expression and AF

**Fig. 3:**
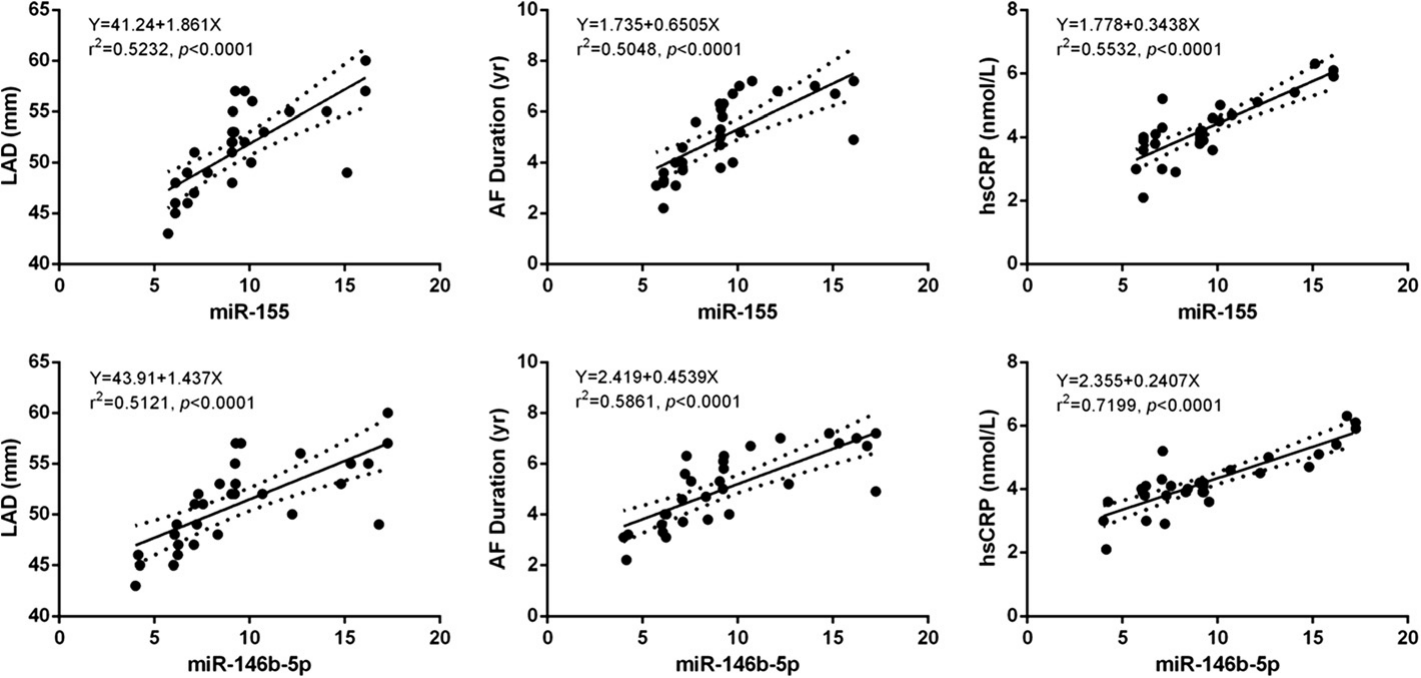
Correlation between expression levels of miRNA-155, miRNA-146b-5p and left atrium dimension, atrial fibrillation duration and high sensitivity C-reactive protein

To determine the factors that influence the levels of miRNAs, we analyzed the association between miRNAs and the baseline characteristics of the patients. There were significant positive correlations between the expression of miR-146b-5p and miR-155, and AF patients’ LAD, plasma levels of high sensitivity C-reactive protein (hsCRP) and AF duration, respectively (Fig. 3). Interestingly, the LAD, AF duration and hsCRP levels are considered to be predictors of increased risk for the AF recurrence [24, 25].

To verify whether the up-regulation of these inflammation-associated miRNAs is the main factor for recurrence of AF, we analyzed the expression of miRNAs in patients with sinus rhythm (SR group) and in patients who had recurrence of AF (AF group) in our original cohort after 2 year follow-up (Additional file 5). As shown in the table, the miRNA-155 and miR-146b expression levels, LAD, hsCRP levels, and AF duration were found to significantly predict recurrence in the univariate regression models. However, in multivariate analysis only miRNA-155 [Hazard Ratio [HR], 1.113; *P* = 0.037) and miR-146b-5p (HR, 1.646; *P* = 0.030) expression levels, LAD (HR, 1.036; *P* = 0.039) and AF duration (HR, 1.216; *P* = 0.044) were found to significantly predict the recurrence of AF.

### MiRNAs expression profile is correlated with genes that are involved in AF

Given the fact that miRNA is involved in post-transcriptional regulation, we endeavored to determine whether there is was correlation between the levels of miRNAs expression and their target genes involved in the pathogenesis of AF. We performed target prediction for miRNAs by using 4 Databases (TargetScan, miRanda, Starbase Clip-seq and miRDB) and identified the genes, based on the similar prediction by at least the 3 of the 4 databases. We found that at least 7 genes were related to AF. Among these, 4 genes were related to the inflammatory response and fibrosis, and three others encoding cardiac ion channel proteins (Additional file 6).

### MRNA and protein expression of target gene

**Fig. 4:**
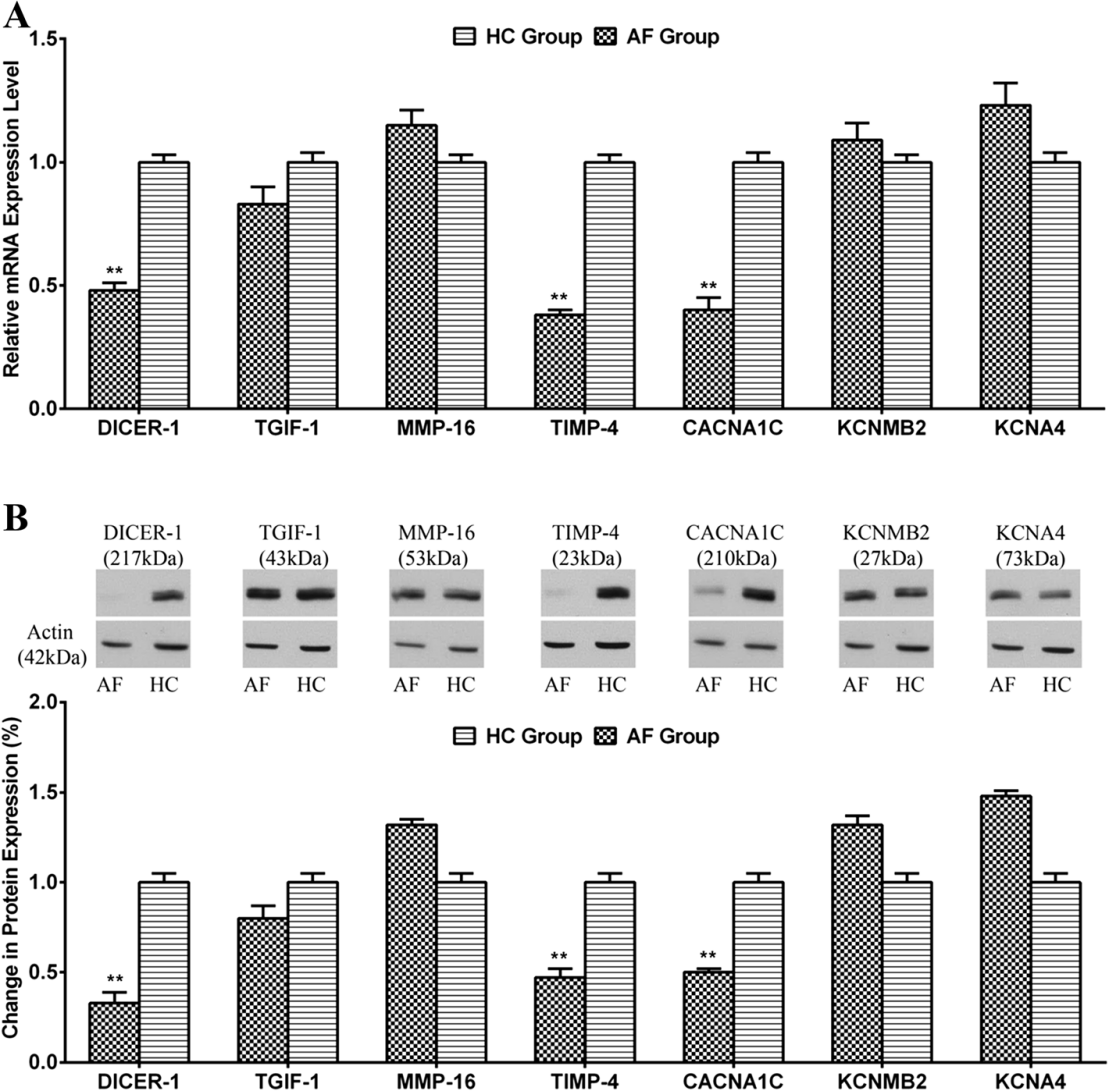
Expression level of target genes in the atrial fibrillation (AF) group (n = 30) and healthy control (HC) group (n = 17). **a** The relative mRNA expression level of the target genes as determined by TaqMan PCR. **b** Protein expression of the target genes using the Western blot. ***P < 0.001; **P < 0.01; *P < 0.05

To determine whether those seven predicted target genes were involved in non-valvular PAF, we measured those expression of mRNA and protein in the entire cohort (n = 47). As a result of TaqMan qPCR, the relative mRNA expression level of the DIER-1, TIMP-4 and CACNA1C were significantly lower in the AF group than that in the HC group (Fig. 4a). As a result of Western blotting analysis, the DIER-1, TIMP-4 and CACNA1C protein expression were also significantly lower in AF group than in HC group (Fig. 4b).

## Discussion

The most important results of our study may be summarized as follows: 1. Expression levels of miR-155, miR-146b-5p and miR-19b were significantly increased in patients with non-valvular PAF compared with HCs. 2. Levels of miR-146b-5p and miR-155, which are known to be associated with inflammation, were independently and positively associated with LAD, AF duration and hsCRP levels. 3. On multivariate analysis only miR-155 and miR-146b-5p expression levels, LAD, and AF duration were found to significantly predict AF recurrence after procedure.

### Co-morbidities might affect miRNAs expression

This study provides the first profiling of miRNAs in patients with non-vavular PAF and HCs. We found that in the LAA miR-155, miR-146b-5p and miR-19b are aberrantly up-regulated in patients with AF compared with HCs. A group of miRNAs regulate genes encoding cardiac ion channel proteins and other relevant genes, and several studies have reported the direct involvement of miRNAs in controlling cardiac excitability and arrhythmogenesis in certain pathological conditions [12]. Some such miRNAs have been shown to be directly involved in AF, and some are considered to have the potential to regulate AF based on their target genes. However, to our knowledge, earlier investigations verified the regulation of miRNAs in the atrial tissue from patients with valvular AF and other co-morbidities in non-AF patients (such as coronary heart disease). Therefore, from those studies it would not possible to deduce whether pre-existing heart diseases already influenced the expression of miRNAs.

Previous studies have reported that expression of miRNAs such as miR-1, miR-328 and miR-499 are altered in valvular AF patients, but it did not significantly change in our study [13–15]. This variation in the findings, compared with other studies, may be attributed to the differences in the tissues that were sampled (LAA in the current study versus left atrial tissue in the other previous studies) and the heterogeneity of human myocardial samples. AF is a multifaceted electrical phenotype involving complex factors beyond electrophysiology and can occur in a variety of pathological settings. The different types of AF have distinct underlying mechanisms, and different miRNAs might be involved. The results of Cooley and Xiao et al. showed that the presence of valvular heart disease in AF influenced miRNA expression patterns in both left and right atria [16, 17].

Morphological and electrophysiological differences have been demonstrated between theright atria and left atria, which at least in part, may reflect different mechanisms involved in AF between the right atria and left atria. Thus, it is not surprising that AF-associated miRNAs of the right atria may differ from those of the left atria. A recent study compared the potential differences of AF-associated miRNAs in the RA and LA from rheumatic mitral valve disease patients. They have found the different distributions of AF-associated miRNAs in the right atrial appendage and LAA from rheumatic mitral valve disease patients. This may reflect different miRNA mechanisms in AF between the RA and LA. 26 But for the non-valvular AF, AF substrate in the left atria is related to AF initiation and maintenance, which plays an important role to initiate and perpetuate AF. And the potential difference of AF-associated miRNAs between right atria and left atria are still unknown due to lack of the RA and LA sample availability.

In our study miR-155, miR-146b-5p, and miR-19b were significantly up-regulated in non-valvular PAF patients. Most important, we also found significantly higher levels of expression of miR-155 and miR-146b-5p were independently associated with LAD, AF duration and hsCRP levels. However, only miR-155 and miR-146b expression level, LA dimension and AF duration were found to significantly predict AF recurrence in multivariate analysis.

### Role of LAA in maintaining AF

Less invasive AF ablation procedures have been developed due to increasing knowledge about the pathophysiology of AF and due to the development of ablation devices, which replace the original ‘cut-and-sew’ technique for scarification [26, 27]. Wolf et al. developed an off-pump procedure, in which the pulmonary veins (PVs) are isolated, ganglionic plexus are ablated, and the LAA is amputated through a bilateral thoracotomy [26]. The LAA is derived from the primordial left atrium, which is formed mainly by the adsorption of the primordial PVs and their branches. This similar embryological origin suggests that the LAA may initiate AF like the PVs. This could explain the potential and active role of LAA as an underestimated source of AF. In our study, miRNAs profiling in LAA provided very compelling evidence, which illustrated the potential of miRNAs as a new mechanism for non-valvular PAF.

### MiRNAs and cardiac remodeling

Interestingly, miR-155, which is reported to be involved in cardiovascular diseases, showed the largest increases in our AF patients compared with HCs. miR-155 might directly influence arrhythmia outcomes by targeting critical ion channel gene expression, including CACNA1C and KCNA4, which encode the cardiac L-type Ca^2+^ channel (I_CaL_) α1c and the initial component of the transient outward potassium current (I_to1_), respectively. And our results showed that the expression of the CACNA1C was significantly lower in the AF group than the HC group, not only for the protein level, but also the mRNA level. Moreover, miR-155 up-regulation may also play a role in inflammation [21]. In fact, emphasis has been given to miR-155 because it represents a typical multifunctional miRNA [28]. Even though the mechanism that regulates miRNA function and expression has not been completely understood, the information presently available (i.e. over expression in AF patients) allows us to recognize miR-155 as a gene of potentially paramount clinical importance in AF diagnosis and treatment. It is reasonable to speculate that evaluation of mRNA-155 in tissues or biological fluids might be utilized as a biochemical parameter for AF detection and prognosis.

MiR-146, is also thought to be a mediator of inflammation along with miR-155 [29]. The expression of miR-146 is up-regulated by inflammatory factors such as interleukin 1 and tumor necrosis factor [22]. miR-146 regulates a number of targets, which are mostly involved in Toll-like receptor pathways that bring about a cytokine response as part of the innate immune system [30]. Recently, miR-146b-5p was reported to be up-regulated in AF patients, which is consistent with our findings [18]. Potential targets of miR-146b-5p include TGIF1, MMP16 and TIMP4 that are reported to be regulated in cardiomyocyte fibrosis, and these might be expected to contribute to AF by promoting fibrosis, an established AF substrate [31]. This possibility remains to be investigated. Our results showed that the expression of the TIMP-4 was significantly lower in the LAA of the AF group than in that of the HC group, not only for the protein level, but also the mRNA level.

MiR-19b also takes part in inflammatory responses enhancing or repressing pro-inflammatory mediators’ expression [23]. It positively regulates Toll-like receptor signaling with DICER-1 deletion and miRNA depletion. The miR-19b is an important protagonist in this phenomenon, regulating positively the NF-kB activity [23]. The miRNA depletion inhibits cytokines production by NF-kB. Also, it is directly involved in the modulation of several NF-kB signaling negative regulators expression, indicating the importance of miR-19b on NF-kB signaling. This miRNA has not been extensively studied, but our data suggests a possible role of this miRNA in the pathology of AF, which is yet to be identified.

### Study limitations

One of the limitations of the present study is that at this point we are unable to provide molecular insights for the cause of miRNA dysregulation in non-valvular PAF. The levels of miRNAs may be affected by multiple factors such as the change in expression in the tissue, the release of the miRNAs by the cells and the stability of miRNAs. Secondly, the exact targets and pathways by which alterations in miRNAs cause AF remain elusive. Although our analysis has indicated some potential genes and pathways that are involved in AF, it is difficult to estimate the actual false-positive rate of the overall target prediction. A better understanding of the biological significance of these broad, but often subtle, changes in miRNA expression that are found in AF patients could be achieved with the development of novel experimental models, in which the levels of one or more miRNAs could be precisely manipulated.

## Conclusions

The present study provides the first insight into the levels of miRNAs in the LAA in patients with non-valvular PAF. While previous studies have demonstrated reliable measurement of miRNAs, our study specifically addressed the levels and regulation of ionic, inflammatory and fibrotic derived miRNAs and shows, specific miRNAs which may be involved in both electrical and structural remodeling of the fibrillating atria. However, these data further need to be confirmed in larger clinical population. Furthermore, the mechanisms underlying the dysregulation, and as well as the putative impact of the changes in miRNAs levels in the physiology or pathophysiology, remains to be elucidated.

## Data availability

The authors confirm that all the supporting data are included as additional files.

## Additional files

Additional file 1:
**Methods.** (DOC 42 kb) Additional file 2:
**Table S1.** Characteristics of study cohort. (DOCX 46 kb) Additional file 3:
**Additional supporting data.** (ZIP 399 kb) Additional file 4:
**Table S2.** Characteristics of miRNAs profiling study cohort. (DOC 36 kb) Additional file 5:
**Table S3.** Cox regression analysis for predictors of atrial fibrillation recurrences. (DOCX 78 kb) Additional file 6:
**Table S4.** The most overrepresented pathways for miRNAs’ targets according to KEGG. (DOCX 44 kb)

## Abbreviations

AF: Atrial fibrillation
FDR: False discovery rate
HCs: Healthy controls
HR: Hazard Ratio
LAA: Left atrial appendage
LAD: Left atrial diameter or dimension
mRNAs: Messenger RNAs
PAF: Paroxysmal atrial fibrillation
QPCR: Quantitative PCR
RT-PCR: Real time PCR
SR: Sinus rhythm
