# Supplementary material for: MicroRNA profiling in the left atrium in patients with non-valvular paroxysmal atrial fibrillation
Source: BMC Cardiovasc Disord. 2015 Aug 29;15:97. doi: 10.1186/s12872-015-0085-2 (PMC4553004; doi:10.1186/s12872-015-0085-2)
Supplement: Additional file 1: — Methods. (DOC 42 kb) [file 12872_2015_85_MOESM1_ESM.doc]

**Supplemental Materials and Methods**

***Study Population***

The study protocol conformed to the ethical guidelines of the 1975 Declaration of Helsinki as reflected in a priori approval by the institution's human research committee. IIt was approved by the Ethics Committee for use of human samples of the Capital Medical University. Thirty patients with PAF according to American Heart Association/American College of Cardiology guidelines were enrolled in this study in Beijing Anzhen Hospital.^[1]^ Patients with prior catheter ablation, severe structural heart disease, and left atrial diameter (LAD) of ≥65 mm or ejection fraction ≤40% were excluded from the study. A written informed consent was obtained from all the subjects. All patients underwent pulmonary vein isolation and exclusion of the LAA using a video-assisted surgical approach.^[2]^ This technique offers a safe and easy approach to remove the LAA. Seventeen dumped LAA specimens obtained from healthy donor hearts without any evidence of AF or cardiovascular diseases during heart transplantation served as healthy controls (HCs). These tissues are considered surgical waste in accordance with Chinese ethical laws.

***Total RNA (including miRNA) Isolation***

Total RNA containing small RNAs were extracted from LAA samples using the Trizol reagent (Invitrogen) and purified with mirVana miRNA Isolation Kit (Ambion, Austin, TX, USA) according to manufacturer’s protocol. Purity and concentration of RNA were determined at an optical density of 260/280 using spectrophotometer (NanoDrop ND-1000，Thermo Scientific, Wilmington, DE). RNA integrity was determined by 1% formaldehyde denaturing gel electrophoresis. RNA samples were stored at -80 °C prior to TaqMan miRNA array studies.

***MiRNA Reverse Transcription and TLDA Quantitative PCR***

Total RNAs were isolated using the mirVana miRNA Isolation Kit (Ambion, Austin, TX, USA). To assess the levels of specific miRNAs in LAA samples, reverse transcription (RT) reaction was carried out according to the company’s recommendations. About 500 ng of miRNAs were reverse transcribed using the Megaplex Primer Pools (Human Pools A and B) from Applied Biosystems. In each array, 3 endogenous controls and a negative control were included for data normalization. The RT reaction system was prepared according to the manufacturer’s recommendations (0.8 μL of 10x Pooled Primers were combined with 0.2 μL of 100 mmol/L dNTPs with dTTP, 0.8 μL of 10x Reverse-Transcription Buffer, 0.9 μL of MgCl_2_ [25 mmol/L], 1.5 μL of Multiscribe Reverse-Transcriptase [50 U/μL] and 0.1 μL of RNAsin [20 U/μL] to a final volume of 7.5 μL). The real time PCR (RT-PCR) reaction was programmed as: 16 °C for 2 min, 42 °C for 1 min and 40 cycles of 50 °C for 1 sec, and then incubation at 85 °C for 5 min using a GeneAmp PCR System 9700 (Applied Biosystems).

After RT-PCR was completed, subsequent miRNA expression profiles were acquired using the low-density miRNA TaqMan array according to the manufacturer’s instructions. The expression profile of miRNAs in each sample was determined using the Human Taqman miRNA Arrays A and B (Applied Biosystems). The PCR reactions were performed using 450 μL of the TaqMan Universal PCR Master Mix No AmpErase UNG (2x) and 6 μL of the reverse transcript product to a final volume of 900 μL. One hundred μL of the PCR mix was dispensed to each port of the TaqMan miRNA Array. The fluidic card was then centrifuged and mechanically sealed. The qPCR was carried out on an Applied Biosystems 7900HT thermocycler under the following recommended conditions: 95° C for 10 min followed by 40 cycles of 95° C for 15 sec and 60° C for 1 min. Raw C_q_ (quantification cycle: the crossing point between the baseline corrected amplification curve and threshold line) values were calculated using the SDS software v.2.3.

***Statistical Analysis on miRNA Data***

Cycle threshold (C_T_) number >37 was considered as non-specific and undetectable; in 95% of individual observations the miRNAs had a C_T_ number >37, and these were excluded from the final data analysis. Significance analysis of the microarrays (SAM, version 3.02) was performed to determine the significance of differentially expressed miRNAs. To select the differentially expressed miRNA genes, we used threshold values of ≥2 and ≤2-fold change, and the false discovery rate (FDR) correction for multiple comparisons of <0.05, to examine the differentially expressed miRNAs compared with the controls. The C_T_ data was centered by genes using the Adjust Data function of CLUSTER 3.0 software, and further analyzed by the hierarchical clustering with average linkage. Finally, we performed the tree visualization by using Java Treeview (Stanford University School of Medicine, Stanford, CA, USA).

***Quantitative Real Time PCR***

For the miRNA, the reverse transcription of RNA was conducted using the TaqMan microRNA Reverse Transcription kit (ABI) according to the manufacturer’s recommendations. Subsequently, 3 μL of the product was used for detecting miRNA expression by quantitative RT-PCR using TaqMan microRNA Assay kits (ABI) for the corresponding miRNA. For the mRNA, the total RNA was reversed transcribed using the M-MLV Reverse Transcriptase (Invitrogen) and random primers. Then the product was used for detecting mRNA expression by quantitative RT-PCR using Power SYBR Green PCR Master Mix (ABI) for the corresponding mRNA. The PCR was conducted using a 7900HT Sequence Detection System (Applied Biosystems, Austin, TX, USA). All of the reactions were run in triplicate. The values of the different miRNAs were normalized to cel-miR-39 and are expressed as 2^− (CT[microRNA]−CT[cel-miR-39])^. The relative expression levels of the mRNAs were calculated based on GAPDH levels and multiplied by 10^2^.

***Western Blot***

Western blotting was performed to determine target genes protein expression. All proteins from frozen LAA samples were resolved on an 8% SDS-denatured polyacrylamide gel and were then transferred onto a nitrocellulose membrane (Micron Separations). Membranes were incubated with the antibody overnight at 4℃. The membranes were washed and incubated with a horseradish peroxidase-conjugated secondary antibody. The reactions were developed with enhanced chemiluminescence reagents (NEN Life Science or Pierce), and the images were obtained by exposure to x-ray films. The films were digitized and quantified with the Image Quant software. All antibodies were purchased from Merck Millipore (MA, USA).

***Statistics***

All results for continuous variables are expressed as mean ± standard error of the mean, and categorical variables are expressed as the number of cases and percentage. The significance of differences between the two groups was assessed either by the Student's *t* test for continuous variables or by the Chi-square test for categorical variables. A Cox regression analysis was used to identify the factors associated with recurrence using those variables with *P* values <0.05 between patients with and without recurrence. Variables selected to be tested in the multivariate analysis were those with a *P* value <0.05 in the univariate models. MiRNAs were log-transformed for the multiple logistic regression model in order to improve linear fitting. Logistic regression analyses were performed to identify variables independently associated with expression levels of miRNAs. All tests were 2-tailed, and a *P-*value <0.05 was considered significant. The data were analyzed with SPSS for Windows software, version 12.0 (SPSS Inc., Chicago, IL, USA).

**References**

1. January CT, Wann LS, Alpert JS, et al. 2014 AHA/ACC/HRS Guideline for the Management of Patients With Atrial Fibrillation: A Report of the American College of Cardiology/American Heart Association Task Force on Practice Guidelines and the Heart Rhythm Society. Circulation 2014.

2. Wang JG, Xin M, Han J, Li Y, Luo TG, Wang J, Meng F, Meng X. Ablation in selective patients with long-standing persistent atrial fibrillation: medium-term results of the Dallas lesion set. Eur J Cardiothorac Surg 2014.
