# Supplementary material for: MicroRNA profiling in the left atrium in patients with non-valvular paroxysmal atrial fibrillation
Source: BMC Cardiovasc Disord. 2015 Aug 29;15:97. doi: 10.1186/s12872-015-0085-2 (PMC4553004; doi:10.1186/s12872-015-0085-2)
Supplement: Additional file 2: — Table S1. Characteristics of study cohort. (DOCX 46 kb) [file 12872_2015_85_MOESM2_ESM.docx]

**Table 1. Characteristics of study cohort**

|  | HCs (n = 17) | PAF (n = 30) | P value |
| --- | --- | --- | --- |
| Gender (male) | 13 | 19 | 0.517 |
| Age (years) | 43 ± 1.3 | 47.3 ± 6.8 | 0.581 |
| NYHA function class | 1.1 ± 0.1 | 2.3 ± 0.2 | 0.203 |
| AF duration (years) | 0 | 4.2 ± 5.1 | 0.001* |
| LAD (mm) | 41.8 ± 2.3 | 53.1 ± 2.2 | 0.003* |
| Concurrent medication |  |  |  |
| Beta-blocker | 0 | 2 | 0.528 |
| Amiodarone | 0 | 5 | 0.143 |
| Digitalis | 0 | 2 | 0.528 |
| Aspirin/Clopidogrel/Warfarin | 0 | 4 | 0.281 |
| hsCRP (nmol/L) | 10.5 ± 4.8 | 47.0 ± 9.6 | 0.001* |

Data are presented as the number (%) of patients or mean ± SD. hsCRP, high sensitivity C-reactive Protein; HC, health control; LAD, left atrium dimension; PAF, paroxysmal atrial fibrillation, AF, atrial fibrillation. *Significant AF group vs. HC group.
