# Supplementary material for: MicroRNA profiling in the left atrium in patients with non-valvular paroxysmal atrial fibrillation
Source: BMC Cardiovasc Disord. 2015 Aug 29;15:97. doi: 10.1186/s12872-015-0085-2 (PMC4553004; doi:10.1186/s12872-015-0085-2)
Supplement: Additional file 3: — Additional supporting data. (ZIP 399 kb) [file 12872_2015_85_MOESM3_ESM.doc]

**Table2 . Characteristics of miRNAs profiling** study cohort

|  | HCs (n = 5) | PAF (n = 8) | P value |
| --- | --- | --- | --- |
| Gender (male) | 3 | 4 | 1.000 |
| Age (years) | 42 ± 1 | 46 ± 1 | 0.931 |
| NYHA function class | 1.0 ± 0.1 | 2.1 ± 0.3 | 0.609 |
| AF duration (years) | 0 | 3.9 ± 4.8 | 0.001* |
| LAD (mm) | 41.2 ± 1.9 | 52.9 ± 3.6 | 0.008* |
| Concurrent medication |  |  |  |
| Beta-blocker | 0 | 1 | 1.000 |
| Amiodarone | 0 | 1 | 1.000 |
| Aspirin/Clopidogrel/Warfarin | 0 | 2 | 0.505 |
| hsCRP (nmol/L) | 9.9 ± 3.1 | 45.2 ± 7.5 | 0.001* |

Data are presented as the number (%) of patients or mean ± SD. hsCRP, high sensitivity C-reactive Protein; HC, health control; LAD, left atrium dimension; PAF, paroxysmal atrial fibrillation, AF, atrial fibrillation. *Significant AF group vs. HC group.
