# Supplementary material for: MicroRNA profiling in the left atrium in patients with non-valvular paroxysmal atrial fibrillation
Source: BMC Cardiovasc Disord. 2015 Aug 29;15:97. doi: 10.1186/s12872-015-0085-2 (PMC4553004; doi:10.1186/s12872-015-0085-2)
Supplement: Additional file 4: — Table S2. Characteristics of miRNAs profiling study cohort. (DOC 36 kb) [file 12872_2015_85_MOESM4_ESM.docx]

**Table 3. Cox regression analysis for predictors of atrial fibrillation recurrences**

| **Variables** | **AF group**  **(n = 7)** | **SR group**  **(n = 23)** | **Univariate Analysis** | | |  | **Multivariate Analysis** | | |
| --- | --- | --- | --- | --- | --- | --- | --- | --- | --- |
|  |  |  | **HR** | **95% CI** | ***P* value** |  | **HR** | **95% CI** | ***P* value** |
| Gender (male) | 4 | 15 | 1.823 | 0.896 - 1.782 | 0.671 |  | 1.214 | 1.012 - 2.692 | 0.832 |
| Age (years) | 48.6 ± 4.9 | 49.0 ± 5.3 | 1.540 | 0.559 - 2.229 | 0.745 |  | 1.634 | 0.952 - 2.806 | 0.767 |
| LAD (mm) | 56.4 ± 3.4* | 45.8 ± 6.9 | 1.036 | 1.017 - 1.094 | 0.024 |  | 1.036 | 1.017 - 1.074 | 0.039 |
| AF duration (year) | 5.8 ± 4.6* | 3.2 ± 2.9 | 1.815 | 1.135 - 2.156 | 0.034 |  | 1.216 | 1.124 - 1.811 | 0.044 |
| hsCRP (nmol/L) | 53.3 ± 12.4* | 30.5 ± 10.5 | 1.343 | 1.023 - 1.960 | 0.042 |  | 1.259 | 1.034 - 2.061 | 0.535 |
| Mir-155, 2^-ΔCT^(*10^-4^) | 5.91 ± 4.73* | 2.45 ± 1.58 | 1.523 | 1.013 - 2.690 | 0.029 |  | 1.113 | 1.118 - 2.801 | 0.037 |
| Mir-146b-5p, 2^-ΔCT^(*10^-4^) | 7.21 ± 4.16* | 5.14 ± 2.47 | 1.587 | 0.893 - 2.810 | 0.017 |  | 1.646 | 1.014 - 2.691 | 0.030 |
| Mir-19b, 2^-ΔCT^(*10^-4^) | 6.48 ± 3.22 | 5.65 ± 2.27 | 1.603 | 1.348 - 1.850 | 0.727 |  | 1.849 | 1.528 - 2.651 | 0.836 |

AF, atrial fibrillation; HR, hazard ratio; CI, confidence interval; hsCRP, high sensitivity C-reactive Protein; LAD, left atrium dimension; SR, sinus rhythm. *Significant AF group vs. SR group.
