# Supplementary material for: MicroRNA profiling in the left atrium in patients with non-valvular paroxysmal atrial fibrillation
Source: BMC Cardiovasc Disord. 2015 Aug 29;15:97. doi: 10.1186/s12872-015-0085-2 (PMC4553004; doi:10.1186/s12872-015-0085-2)
Supplement: Additional file 5: — Table S3. Cox regression analysis for predictors of atrial fibrillation recurrences. (DOCX 78 kb) [file 12872_2015_85_MOESM5_ESM.docx]

**Table 4. The most overrepresented pathways for miRNAs’ targets according to KEGG.**

|  | **KEGG Pathway Term** | **Count** | ***P* value** | **Gene Names** |
| --- | --- | --- | --- | --- |
| Inflammation and fibrosis | Toll-like receptor signaling pathway  TGF-beta signaling pathway  TNF signaling pathway | 1  2  1 | 1.34e-04  1.50e-02  1.42e-02 | DICER-1  TGIF1  MMP-16 TIMP-4 |
| Ion transport |  | 3 | 4.32e-02  3.63e-02  2.09e-02 | CACNA1C  KCNMB2  KCNA4 |
