# Supplementary figures and images for: MicroRNA profiling in the left atrium in patients with non-valvular paroxysmal atrial fibrillation
Source: BMC Cardiovasc Disord. 2015 Aug 29;15:97. doi: 10.1186/s12872-015-0085-2 (PMC4553004; doi:10.1186/s12872-015-0085-2)

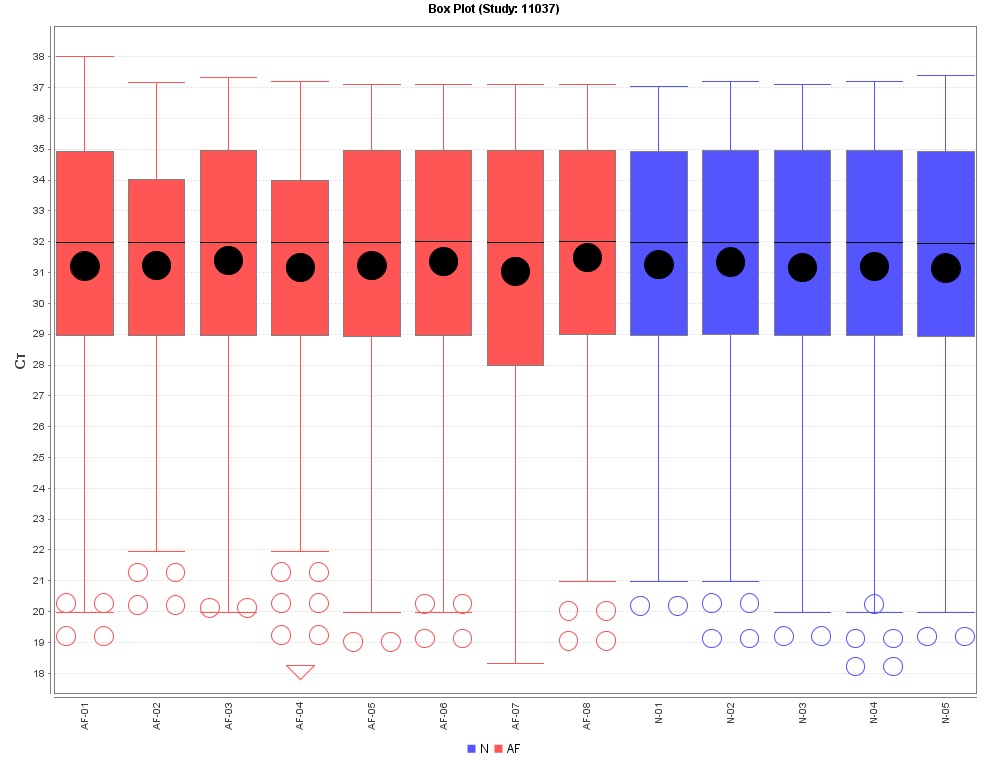

Supplement: Additional file 6: — Table S4. The most overrepresented pathways for miRNAs’ targets according to KEGG. (DOCX 44 kb) [file 12872_2015_85_MOESM6_ESM.zip › Additional Supporting DATA/Box Plot/Box Plot.jpg]

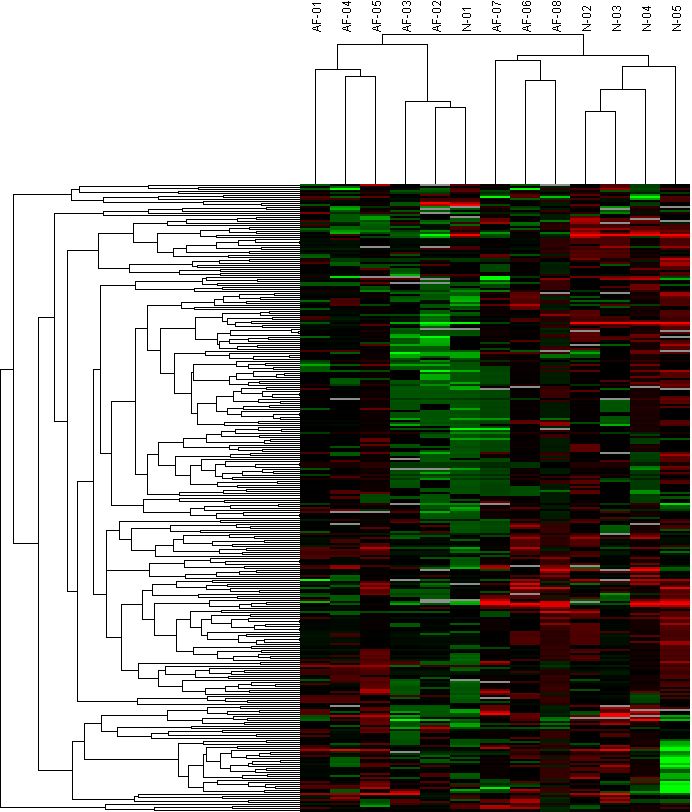

Supplement: Additional file 6: — Table S4. The most overrepresented pathways for miRNAs’ targets according to KEGG. (DOCX 44 kb) [file 12872_2015_85_MOESM6_ESM.zip › Additional Supporting DATA/Cluster data/cluster.jpg]

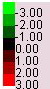

Supplement: Additional file 6: — Table S4. The most overrepresented pathways for miRNAs’ targets according to KEGG. (DOCX 44 kb) [file 12872_2015_85_MOESM6_ESM.zip › Additional Supporting DATA/Cluster data/cluster_colorbar.jpg]
